# Heat, heatwaves, and ambulance service use: a systematic review and meta-analysis of epidemiological evidence

**DOI:** 10.1007/s00484-023-02525-0

**Published:** 2023-07-27

**Authors:** Zhiwei Xu, Jessica T. Watzek, Dung Phung, Mehak Oberai, Shannon Rutherford, Aaron J.E. Bach

**Affiliations:** 1 School of Medicine and Dentistry, Griffith University, Parklands Drive, Southport, Gold Coast, QLD 4222 Australia; 2 Cities Research Institute, Griffith University, Gold Coast, Australia; 3 School of Public Health, The University of Queensland, Brisbane, Australia

**Keywords:** Emergency medical services, Heat stress, Heat-related illness, Excess heat factor, Heatwave intensity

## Abstract

Ambulance data has been reported to be a sensitive indicator of health service use during hot days, but there is no comprehensive summary of the quantitative association between heat and ambulance dispatches. We conducted a systematic review and meta-analysis to retrieve and synthesise evidence published up to 31 August 2022 about the association between heat, prolonged heat (i.e. heatwaves), and the risk of ambulance dispatches. We initially identified 3628 peer-reviewed papers and included 48 papers which satisfied the inclusion criteria. The meta-analyses showed that, for each 5 °C increase in mean temperature, the risk of ambulance dispatches for all causes and for cardiovascular diseases increased by 7% (95% confidence interval (CI): 5%, 10%) and 2% (95% CI: 1%, 3%), respectively, but not for respiratory diseases. The risk of ambulance dispatches increased by 6% (95% CI: 4%, 7%), 7% (95% CI: 5%, 9%), and 18% (95% CI: 12%, 23%) under low-intensity, severe, and extreme heatwaves, respectively. We observed two potential sources of bias in the existing literature: (1) bias in temperature exposure measurement; and (2) bias in the ascertainment of ambulance dispatch causes. This review suggests that heat exposure is associated with an increased risk of ambulance dispatches, and there is a dose-response relationship between heatwave intensity and the risk of ambulance dispatches. For future studies assessing the heat-ambulance association, we recommend that (1) using data on spatially refined gridded temperature that is either very well interpolated or derived from satellite imaging may be an alternative to reduce exposure measurement bias; and (2) linking ambulance data with hospital admission data can be useful to improve health outcome classification.

**Supplementary Information:**

The online version contains supplementary material available at 10.1007/s00484-023-02525-0.

## Introduction

High ambient temperatures (i.e. heat) can adversely impact human health; including but not limited to the circulatory (Liu et al. 2022), respiratory (Cheng et al. 2019), renal (Liu et al. 2021a), nervous (Liu et al. 2021b), and endocrine systems (Moon 2021). Even in advanced economies, extreme heat is the most deadly natural hazard (Borden and Cutter 2008). Extreme heat, which already causes US $727 billion in economic losses worldwide each year (Callahan and Mankin 2022), is becoming more frequent and intense due to climate change.

Multi-national studies have reported an increased risk of mortality associated with heat (Guo et al. 2018; Vicedo-Cabrera et al. 2018). The effect of heat on healthcare services (e.g. hospital admissions) has previously been meta-analysed (Phung et al. 2016). Some recent studies have found that dispatch call centres and ambulances can more readily detect the acute effects of heat (Wang et al. 2021; Xu et al. 2018). Compared with emergency department presentation and hospital admission data, ambulance data allows for a more sensitive indication of total demand for health services allowing for better preparation, management, and deployment during heat events (Bassil 2010). It is worthwhile synthesising published evidence on heat and ambulance service use.

We systematically reviewed and summarised evidence provided in the available literature on heat, prolonged heat (i.e. heatwaves) and ambulance service use, aiming to (1) provide a pooled effect estimate for the impacts of heat and heatwaves on all-cause and cause-specific ambulance dispatches; and (2) identify potential sources of bias in the study methodology.

## Materials and methods

This review was conducted in alignment with the most up-to-date version of Preferred Reporting Items for Systematic Review and Meta-Analysis (PRISMA) Guidelines (Page et al. 2021), and a protocol was registered through the international prospective register of systematic reviews (PROSPERO Reference: CRD42022296556).

### Eligibility criteria

Empirical studies were considered eligible for inclusion within the review if they met the following criteria: (1) human population; (2) English language; (3) published within the last decade (2011–2022); (4) included a measure of heat or heatwaves as the primary exposure; (5) used routinely collected ambulance records to investigate outcomes; (6) the outcome was public health-related (e.g. not performance assessment of ambulance services, nor occupational based). In this review, we defined heat as temperatures above an optimal temperature range or optimal temperature point which have adverse impacts on human health and well-being (Asseng et al. 2021; Gasparrini et al. 2015). As this review focused on the impact of short-term exposure to heat or heatwaves on the risk of ambulance dispatches, we included studies with the two most common designs which quantify the association between short-term exposure to heat or heatwaves and risk of health outcomes: time-series and case-crossover designs (Wu et al. 2022).

There were three exclusion criteria: (1) any studies relying exclusively on hospital, death registry, or emergency department admission records were excluded; (2) as the primary exposure of interest was heat and heatwaves, if any study investigated the impact of air pollution as the primary exposure, and included temperature as a confounding exposure, then these studies were also excluded. Temperature is a potential confounder of the association between short-term exposure to air pollution (particularly ozone) and the risk of health outcomes. For instance, the concentrations of ozone tend to increase during hot weather, and the association between short-term exposure to ozone and the risk of health outcomes may be confounded by heat (Alari et al. 2023). Air pollution has been found to be a modifier of the association between short-term heat exposure and the risk of health outcomes (Hu et al. 2022); (3) any study where the performance or evaluation of ambulance and emergency services occurred without evaluation of the public health impacts of heat again were excluded.

### Information sources

After consultation with a research librarian at Griffith University, a search strategy was developed and applied to the following six online databases: PubMed, Embase, Cumulative Index of Nursing and Allied Health Literature (CINHAL), Scopus, ProQuest, and Web of Science. The search strategy consisted of three major themes: (i) a heat term, (ii) an ambulance term, and (iii) a health term. Terms were adjusted to each database using mesh terms and filters where applicable (Supplementary 1). Pilot searches were conducted to ensure search strategy robustness and inclusion of key literature, the final search was conducted on August 31, 2022. The references of identified articles were screened to make sure all relevant articles were included.

### Literature selection and quality assessment

All study results acquired from the six databases searched were imported into Endnote (version X9, 2013), and duplicates were removed before being uploaded into Covidence (v2715, 2021) to complete data extraction and quality assessments. All study titles, abstracts, and full texts were screened by two independent reviewers (JW, and AB, SR, DP, or ZX) using standardised criteria. Any disagreements were resolved via discussion between both reviewers, and if consensus could not be reached, a third reviewer was introduced.

Quality assessment analyses were conducted by two reviewers (JW, and AB, SR, DP, or ZX) using the Newcastle-Ottawa Scale for assessing the quality of nonrandomised studies. The tool was adapted to suit each of the included study designs (time-series and case-crossover). Quality assessment analyses aided in assessing the quality of evidence presented within each study by examining sample representativeness, ascertainment of exposure and outcome measures, inclusion of common confounders within the statistical models used, and the specificity of the outcome presented (Supplementary 2).

### Meta-analysis

For studies assessing the impact of heat on ambulance dispatches, if they used the same temperature indicator (e.g. mean temperature) and health outcome (e.g. ambulance dispatches for cardiovascular diseases), they were included in the same meta-analysis. The definitions of different temperature indicators used in the included studies are presented in the Supplementary 3. Twenty-three studies were excluded from the meta-analysis mainly due to the temperature indicators used:

Six studies used mean temperature as the temperature indicator (Cheng et al. 2016; Hu et al. 2020; Kotani et al. 2018; Onozuka and Hagihara 2015; Prichard et al. 2022; Wu et al. 2021). However, Cheng et al. used warm season temperature to examine the overall effect of heatwaves, Hu et al. used warm season temperature to examine the main and added effects of heatwaves, and Kotani et al., Onozuka and Hagihara, Prichard et al., and Wu et al. did not provide information on the specific values of the temperature cut-offs to define heat. Hence, the effect estimates published in these studies could not be pooled together.

Four studies used apparent temperature (Alessandrini et al. 2011; Hartz et al. 2013; Ng et al. 2014; Pourshaikhian et al. 2019). However, Alessandrini et al. used mean apparent temperature and dichotomised mean apparent temperature into “25 °C to 30 °C” and “> 30 °C”, Hartz et al. used maximum apparent temperature as the temperature indicator and heat-related illnesses as the health outcome, Ng et al. used maximum 3-h apparent temperature as the temperature indicator and heatstroke as the health outcome, and Pourshaikhian et al. used apparent temperature as the temperature indicator and cardiovascular diseases as the health outcomes.

Four studies used local heatwave definitions which were different from each other (Loughnan et al. 2014; Schaffer et al. 2012; Williams et al. 2011; Williams et al. 2020).

Three studies used maximum temperature (Romani et al. 2020; Turner et al. 2013; Williams et al. 2012). However, Romani et al. did not provide information on the values of the 95th percentile (i.e. heat definition) and used cardiovascular diseases as the health outcome, Turner et al. assessed the main and added effects of heatwaves on the risk of cause-specific ambulance dispatches, and Williams et al. assessed the association between maximum temperature and ambulance dispatches for all causes.

Two studies used heat index (Mathes et al. 2017; Zottarelli et al. 2021). However, Mathes et al. examined the association between heatwave and heat-related ambulance dispatches, and Zottarelli et al. assessed the association between heat and ambulance dispatches for all causes.

Two studies used excess heat factor (EHF) (Hatvani-Kovacs et al. 2016; Patel et al. 2019). However, the EHF definitions they used were different from each other, and they were different from the EHF definition used in the other two studies included in the meta-analysis (Jegasothy et al. 2017; Williams et al. 2018).

**Fig. 1:**
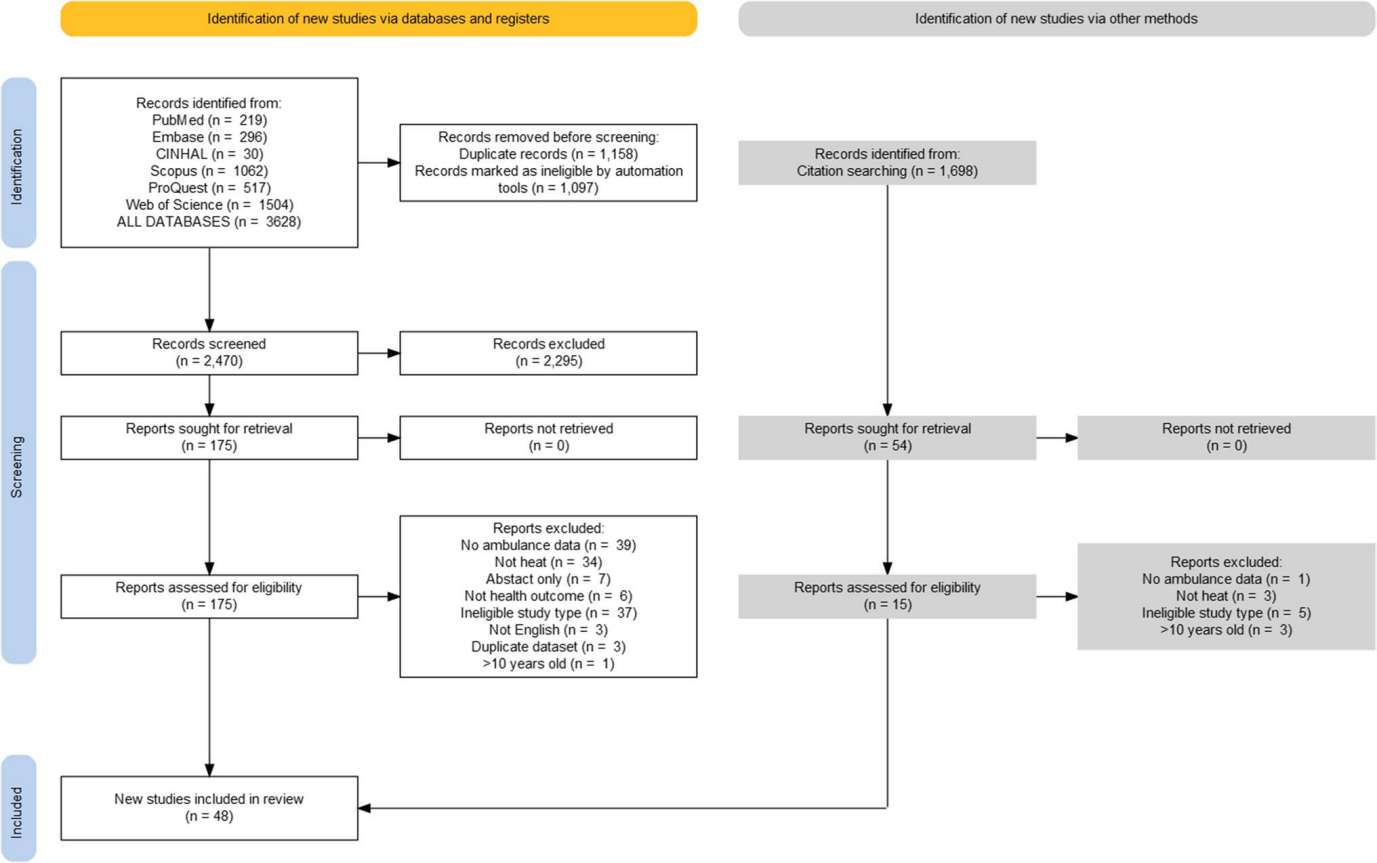
The PRISMA diagram for literature selection flow

One study used humidex as the temperature indicator (Calkins et al. 2016). Fig. 1

One study used compound heat as the temperature indicator (He et al. 2021).

Although there were studies using the same temperature indicator and out-of-hospital cardiac arrest (OHCA), we did not conduct a meta-analysis for OHCA because a separate meta-analysis has been published (Wu et al. 2023). We made two assumptions in the meta-analysis:

We assumed that odds ratio (OR) is a reasonable approximation of relative risk (RR) in the eligible studies because ambulance dispatches are small probability events (i.e. < 1%). Four eligible studies used RR as the effect estimate indicator (Cui et al. 2020; Sangkharat et al. 2020; Wang et al. 2021; Zhan et al. 2018), and Guo’s study used OR (Guo 2017). We assumed OR is a reasonable approximation of RR in Guo’s study. The study of Turner et al. used “percentage change in the risk of ambulance dispatches”, and we also converted it into RR.

We assumed that the association between heat and risk of ambulance dispatches is linear, acknowledging that the association between temperature (i.e. heat and cold) and health outcomes could be U-, J-, or V-shaped. Hence, we converted the RRs under different temperature increments reported in all six eligible studies (Cui et al. 2020; Guo 2017; Sangkharat et al. 2020; Turner et al. 2012; Wang et al. 2021; Zhan et al. 2018) into RRs per 5 °C increase in temperature. This approach has been used elsewhere (Chersich et al. 2020). The linear association between heat and risk of ambulance dispatches in the six eligible studies was shown in the figures of the six published papers (see Figure 2 of Cui et al. 2020, Figure 3 of Guo et al. 2017, Figure 3 of Sangkharat et al. 2020 (mainly for one ambulance dispatch indicator ‘999 ambulance’ but slightly different for the other indicator ‘Red ambulance’), Figure 2 of Wang et al. 2021, and Figure 2 of Zhan et al. 2018 (at lag 0)).

**Fig. 2:**
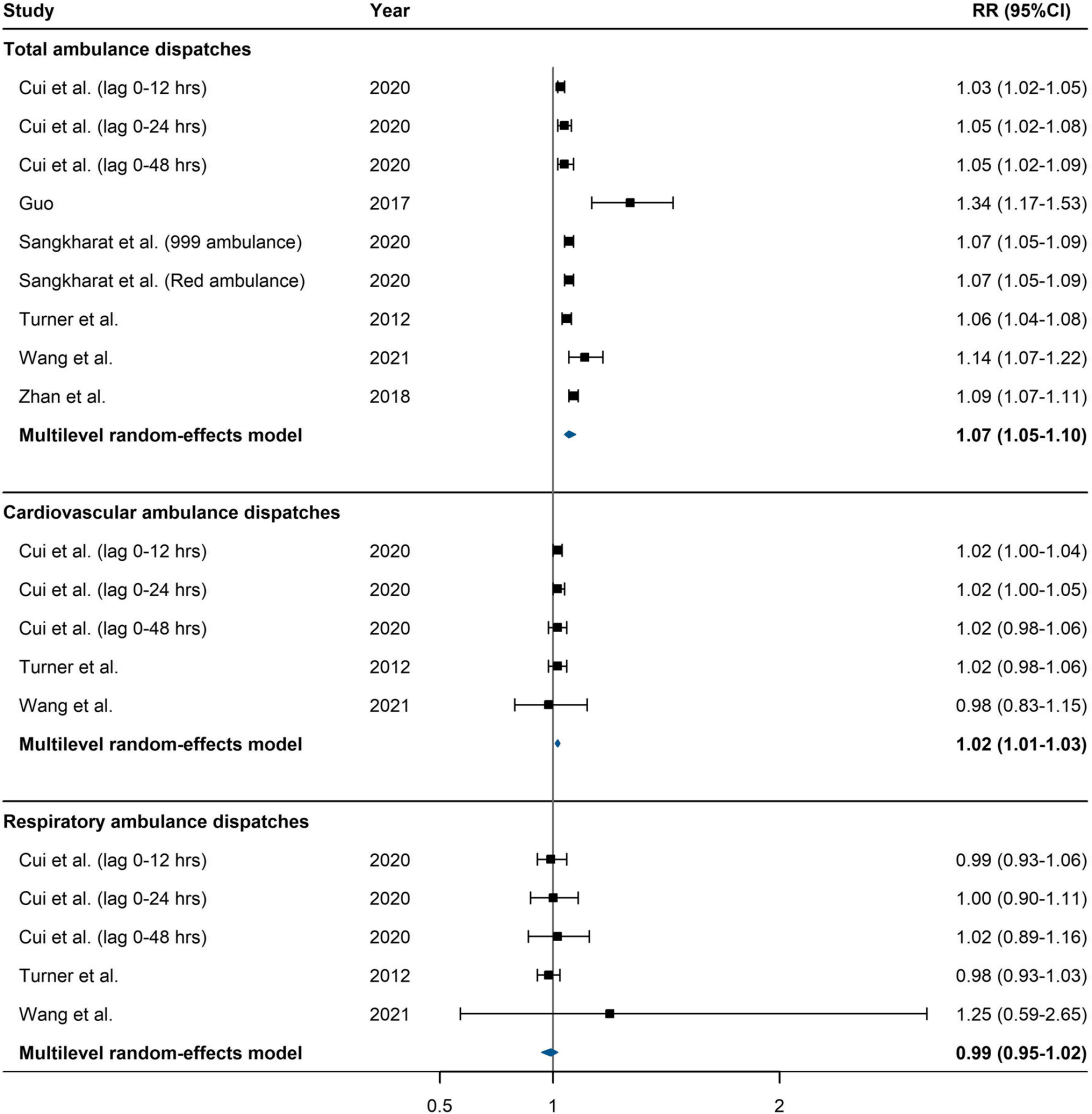
Forest plot for the association between heat and risk of ambulance dispatches

**Fig. 3:**
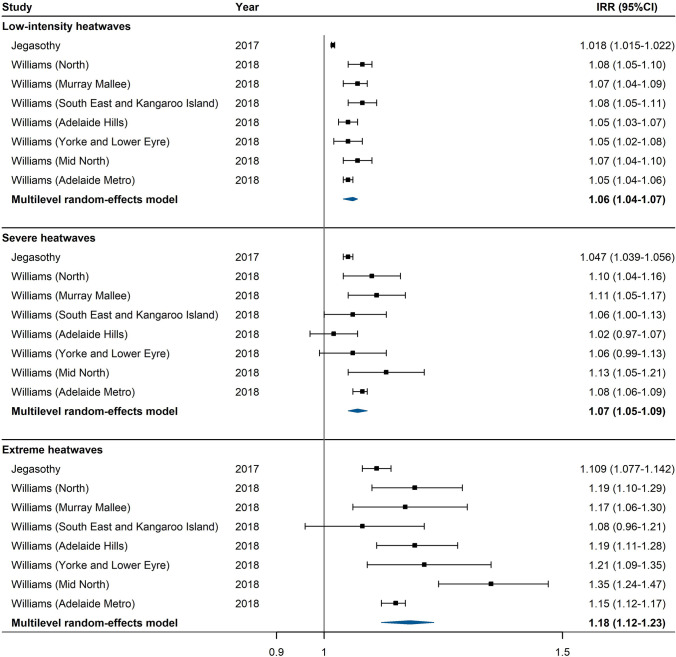
Forest plot for the association between heatwaves (defined by excess heat factor) and risk of ambulance dispatches

The impact of heat on ambulance dispatches may last for more than 1 day (i.e. lagged) but is generally acute. Cui et al. reported RRs across multiple lags (from lag 0–12 h to lag 0–170 h-) (Cui et al. 2020), and Sangkharat et al. reported RRs from 0–2 days to 0–21 days (Sangkharat et al. 2020). We used RRs for lags within 2 days in the meta-analysis because they generally represented the most acute impact of heat (Thomas et al. 2021; Winquist et al. 2016). The acute impact of heat occurred within 2 days was shown in the tables of the three published papers (see Table 2 of Cui et al. 2020, Table 3 of Sangkharat et al. 2020, and Table 3 of Turner et al. 2012).

For studies assessing the impact of heatwaves on ambulance dispatches, if they used the same heatwave definition (e.g. Excess Heat Factor (EHF)), effect estimate indicator (e.g. incidence rate ratio (IRR)), and health outcome (e.g. the daily total number of ambulance dispatches), they were included in the same meta-analysis. Specifically, two Australian studies which used EHF, in the same way, were pooled together (Jegasothy et al. 2017; Williams et al. 2018), and another two studies which used the same heatwave intensity (90th, 95th, or 99th percentiles) and duration indicators (2 or 3 days) were pooled together (Sun et al. 2014; Xu et al. 2018).

Random-effects models were used to pool the effect estimates. For studies which reported RRs across multiple lag periods within 2 days (e.g. 0–12, 0–24, and 0–48 h) (Cui et al. 2020), multiple regions (Williams et al. 2018), or multiple ambulance dispatch indicators (Sangkharat et al. 2020), we used hierarchical random-effects models to pool their RRs with RRs in other studies, accounting for both within-study variability (first level) and between-study variability (second level).

As the number of studies included in each meta-analysis was low (≤ 6), we were unable to assess a funnel plot or do more advanced regression-based assessments to evaluate publication bias.

## Results

### Study selection

The PRISMA diagram in Fig. 1 describes the process of record selection for both the initial database searches and bibliographic screening. The final search yielded 3628 results from the six databases. Following title and abstract screening, this was reduced to 144 studies. After full-text and reference screenings, 48 full texts have been included in this review.

### Quality assessments

Tables 1 and 2 display the quality assessment results for time-series (*n* = 42) and case-crossover studies (*n* = 6), respectively. Four of the included studies (*n* = 48) were of high quality, and the remaining 44 studies were of moderate quality. Among the four high-quality studies, three were of time-series design and one of case-crossover design.

**Table 1:**
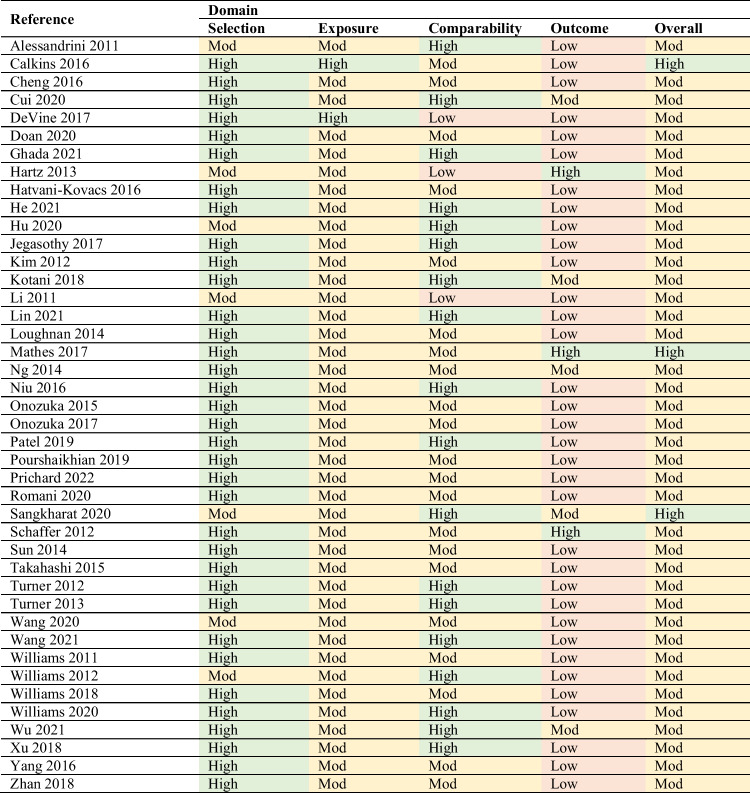
Quality assessment results of time-series studies (*n* = 42)

**Table 2:**
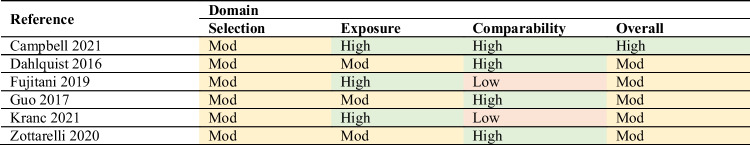
Quality assessment results of case-crossover studies (*n* = 6)

### Study characteristics

Table 3 displays the individual study characteristics and methodological parameters for all 48 studies. Among all the countries/regions, Australia had the highest number of included studies (*n* = 14) (Campbell et al. 2021; Doan et al. 2021; Guo 2017; Hatvani-Kovacs et al. 2016; Jegasothy et al. 2017; Loughnan et al. 2014; Patel et al. 2019; Schaffer et al. 2012; Turner et al. 2012, 2013; Williams et al. 2011; Williams et al. 2012; Williams et al. 2018; Xu et al. 2018), followed by mainland China (*n* = 11) (Cheng et al. 2016; Cui et al. 2020; He et al. 2021; Hu et al. 2020; Li et al. 2011; Niu et al. 2016; Sun et al. 2014; Wang et al. 2021; Wu et al. 2021; Yang et al. 2016; Zhan et al. 2018), Japan (n=6) (Fujitani et al. 2019; Kotani et al. 2018; Ng et al. 2014; Onozuka and Hagihara 2015, 2017; Takahashi and Shimadzu 2015), USA (*n* = 6) (Calkins et al. 2016; Hartz et al. 2013; Mathes et al. 2017; Williams et al. 2020; Zottarelli et al. 2021; DeVine et al. 2017), Taiwan (*n* = 2) (Lin et al. 2021; Wang et al. 2020), UK (*n* = 2) (Prichard et al. 2022; Sangkharat et al. 2020), Germany (*n* = 1) (Ghada et al. 2021), Iran (*n* = 1) (Pourshaikhian et al. 2019), Israel (*n* = 1) (Kranc et al. 2021), Italy (*n* = 1) (Alessandrini et al. 2011), Korea (*n* = 1) (Kim et al. 2012), Spain (*n* = 1) (Romani et al. 2020), and Sweden (*n* = 1) (Dahlquist et al. 2016).

**Table 3:** Descriptive information of included studies (*n* = 48)

Study	City, Country/ Region	Age group and subgroups	Study design and period	Temperature indicator	Adjusted variables	Lag time	Data extracted	Call out reason	Included in the meta-analysis and reason
Alessandrini 2011^*^	Emilia–Romagna, Italy	≥ 35 (35–64, 65–74, ≥ 75)	Time-series 2002–2006	Apparent temperature	NO_2_, O_3_, PM_10_, seasonality, long-term trend, weekends, public holidays	15 days	Location, time, age, sex, urgency, disease code, crude call out number	Non-traumatic causes, cardiosvacular disease (CVD), respiratory disease (RSD)	No, temperature indicator used, temperature variable was dichotomized
Calkins 2016	King County, USA	All ages (0–4, 5–14, 15–44, 45–64, 65–84, ≥ 85)	Time-series 2007–2012	Humidex	Seasonality, long-term trend	-	Call out reason, age, sex, level of transportation, crude call out number	All causes	No, temperature indicator used
Campbell 2021^†^	Tasmania, Australia	All ages (0–5, 0–15, 16–65, >65)	Case-crossover 2008–2019	Excess heat factor	PM_2.5_, public holidays	-	Age, sex, health assessment by paramedic; socioeconomic index for areas, crude call out number	Cardiovascular, respiratory, renal, diabetic, psychological, direct heat-related and other heat-related conditions	No, same heatwave definition as Jegasothy 2017 but different effect estimate indicators
Cheng 2016^†*^	Huainan, China	All ages	Time-series 2011–2013	Mean temperature	Relative humidity, within-season variation, long-term trend, day of the week, public holidays	21 days	Crude call out number only	All causes	No, only warm season data was used in the calculation of heatwave intensity cut-offs
Cui 2020^*^	Luoyang, China	All ages	Case-crossover 2014–2016	Hourly temperature	NO_2_, O_3_, PM_2.5,_ SO_2_	170 hours	Time, disease type, crude call out number	All natural causes, CVD, RSD	Yes
Dahlquist 2016^*^	Stockholm County, Sweden	All ages	Case-crossover 2000–2010	Mean temperature	Relative humidity, O_3_, PM_10_	6 days	Time, health assessment by paramedic, crude call out number	Out-of-hospital cardiac arrest (OHCA)	No, OHCA as the health outcome
DeVine 2017	King County, USA	All ages	Time-series 2007–2012	Humidex	Day of week	-	Call out reason, location, crude call out number	All causes	No, essentially the same dataset as Calkins 2016
Doan 2020^†*^	Brisbane, Australia	All ages	Time-series 2007–2019	Mean temperature	Diurnal temperature range, relative humidity, seasonality, long-term trend	21 days	Crude call out number only	OHCA	No, OHCA as the health outcome
Fujitani 2019^*^	Tottori, Japan	All ages (0–17, 18–64, ≥ 65)	Case-crossover 2017	Maximum air temperature	-	-	Time, age, sex, medical condition, initial diagnosis, crude call out number	Heat stroke	No, temperature indicator and health outcome used
Ghada 2021^*^	Munich, Germany	All ages	Time-series 2014–2018	Mean temperature	Sunshine and relative humidity	3 days	Time, age, sex, health assessment by paramedic, crude call out number	All causes	No, regression analyses were conducted by season
Guo 2017	Brisbane, Australia	All ages (<15, 15–34, 35–64, ≥ 65)	Case-crossover 2001–2007	Hourly temperature	Relative humidity, PM_10_, NO_2_, O_3_	240 hours	Time, age, sex, health assessment by paramedic, crude call out number	All natural causes (i.e., non-accidental causes)	Yes
Hartz 2013^*^	Chicago and Phoenix, USA	All ages	Time-series 2003–2006	Maximum temperature, minimum temperature, apparent maximum temperature	-	-	Location, time, crude call out number	Heat event classification	No, temperature indicator and health outcome used
Hatvani-Kovacs 2016^†*^	Adelaide, Australia	All ages	Time-series 2008–2014	EHF	-	-	Urgency of call, crude call out number	All causes	No, EHF definition used was different from Campbell 2021 or Jegasothy 2017
He 2021^*^	Shenzhen, China	All ages (0–17, 18–44, 45–59, ≥ 60)	Time-series 2015–2016	Compound heat	Relative humidity, SO_2_, O_3_, PM_2.5_, seasonality, long-term trend, day of the week	1 day	Call out reason, age, sex, crude call out number	All causes	No, temperature indicator used
Hu 2020^†^	Shenzhen, China	All ages	Time-series 2013–2017	Mean temperature	Relative humidity, day of the week, public holidays (air pollutants in the sensitivity analyses)	7 days	Call out reason, location, date, time, age, sex, symptoms, primary diagnosis, chief complaint, crude call out number	All causes	No, only used warm season data and separately assessed the main and added effects of heatwaves
Jegasothy 2017^†^	New South Wales, Australia	All ages	Time-series 2005–2015	EHF	Public holidays	-	Crude call out number only	All causes	Yes
Kim 2012	Seven metropolitan areas, South Korea	All ages (< 40, 40–64, ≥ 65)	Time-series 2006–2007	Mean temperature	Relative humidity, long-term trend, day of the week, public holidays	-	Call out reason, crude call out number	Injury	No, injury used as the health outcome
Kotani 2018	Fukuoka, Japan	All ages (0–19, 20–39, 40–59, 60–79, ≥ 80)	Time-series 2005–2012	Mean temperature	Relative humidity, PM2.5, weekdays, public holidays	7 days	Call out reason, time, age, sex, initial diagnosis by doctor at hospital, crude call out number	All causes	No, no information on the specific values of 85^th^ and 95^th^ percentiles
Kranc 2021	Israel	All ages (19–70, ≥ 70)	Case-crossover 2016–2017	Mean temperature	Relative humidity	72 hours	Location, time, age, sex, survival, crude call out number	OHCA	No, OHCA as the health outcome
Li 2011	Beijing, China	All ages	Time-series 2005–2007	Mean temperature	Relative humidity, wind speed	-	Call out reason, health assessment by paramedic, general information, medical history, crude call out number	Acute coronary syndrome	No, acute coronary syndrome as the health outcome
Lin 2021^*^	Kaohsiung, Taiwan	All ages	Time-series 2006–2010	Mean temperature	Relative humidity, PM_2.5_, wind speed, seasonality and long-term trend, day of the week, public holidays	5 days	Crude call out number only	Respiratory distress, coma, unconsciousness, chest pain, headaches, dizziness, vertigo, falling, syncope, lying at public, out-of-hospital cardiac arrest	No, health outcomes too specific
Loughnan 2014	Nine cities, Australia	All ages	Time-series 2000–2011	Mean temperature, maximum temperature	Seasonality, long-term trend	-	Crude call out number only	All causes	No, used local extreme heat definition
Mathes 2017^*^	New York City, USA	All ages	Time-series 1999–2013	Maximum heat index	-	3 days	Location, time, hospital, crude call out number	Heat-related	No, used local extreme heat definition
Ng 2014^*^	Kanto area, Japan	All ages	Time-series 2000–2009	Maximum three-hour apparent temperature	Seasonality, long-term trend, weekends, public holidays	1 day	Call out reason, crude call out number	Heatstroke	No, temperature indicator and health outcome used
Niu 2016	Guangzhou, China	All ages	Time-series 2008–2012	Mean temperature	Seasonality, long-term trend, day of the week, public holidays, relative humidity, PM_10_, NO_2_, SO_2_	21 days	Call out reason, crude call out number	OHCA	No, OHCA as the health outcome
Onozuka 2015	47 prefectures, Japan	All ages	Time-series 2007–2010	Mean temperature	Seasonality, long-term trend, day of the week, public holidays	21 days	Call out reason, cause of the disease according to the ICD10, crude call out number	All causes, CVD, RSD	No, no information on the specific values of the reference temperature
Onozuka 2017^*^	47 prefectures, Japan	> 17 years	Time-series 2005–2014	Mean temperature	Seasonality, long-term trend, day of the week, public holidays	21 days	Crude call out number only	OHCA	No, OHCA as the health outcome
Patel 2019^†^	Perth, Australia	All ages (0–14, 15–59, ≥ 60)	Time-series 2006–2015	EHF	Seasonality, long-term trend, air pollutants	14 days	Call out reason, age, sex, statistical area level, crude call out number	All causes	No, EHF definition was different from the above three EHF studies
Pourshaikhian 2019^*^	Rasht, Iran	All ages (0–64, ≥ 65)	Time-series 2010–2015	Apparent temperature	Seasonality, long-term trend, day of the week, public holidays	20 days	Age, sex, health assessment by paramedic, crude call out number	CVD	No, temperature indicator used
Prichard 2022^*^	Three cities, UK	All ages	Time-series 2007–2017	Mean temperature	Seasonality, long-term trend, day of the week, public holidays	21 days	Crude call out number only	All causes	No, no information on the specific values of 95^th^ percentile
Romani 2020	Two cities, Spain	All ages	Time-series 2005–2017	Minimum and maximum temperatures	Seasonality, long-term trend	14 days	ICD code, crude call out number	CVD	No, no information on the specific values of 95^th^ percentile
Sangkharat 2020^*^	London, UK	All ages	Time-series 2010–2014	Mean temperature	Seasonality, long-term trend, day of the week, public holidays, relative humidity, influenza	21 days	Call out reason, urgency of call, crude call out number	CVD, RSD, non-cardiorespiratory categories	Yes
Schaffer 2012^†^	Sydney, Australia	All ages (< 75, ≥ 75)	Time-series 2006–2011	Minimum and maximum temperatures	Seasonality, long-term trend, day of the week, public holidays	4 days	Call out reason, crude call out number	Heat-related	No, used local heatwave definition
Sun 2014^*^	Pudong New Area, Shanghai, China	All ages	Time-series 2011–2013	Mean temperature	Seasonality, long-term trend, day of the week, relative humidity	7 days	Crude call out number only	All causes	Yes
Takahashi 2015^*^	Japan	All ages	Time-series 2005–2011	Mean temperature	Seasonality, long-term trend, day of the week	6 days	Crude call out number only	OHAC	No, OHAC as the health outcome
Turner 2012^*^	Brisbane, Australia	All ages	Time-series 2000–2007	Mean temperature	Seasonality, long-term trend, day of the week, PM_10_, O_3_, NO_2_, SO_2_	27 days	Location, age, sex, health assessment by paramedic, crude call out number	All causes, CVD, RSD, non-cardiorespiratory categories	Yes
Turner 2013^†^	Brisbane, Australia	All ages (15–64, 65–74, ≥ 75)	Time-series 2000–2007	Maximum temperature	Seasonality, long-term trend, day of the week, PM_10_, O_3_, NO_2_	10 days	Call out reason, location, age, sex, health assessment by paramedic, crude call out number	All causes, CVD, RSD	No, separately assessed the main and added effects of heatwaves, but used year-round data (different from Hu 2020)
Wang 2020	15 cities/counties, Taiwan	All ages	Time-series 2006–2014	Mean temperature	Long-term trend, day of the week, public holidays, particulate matter, NO_2_, wind speed, relative humidity, pneumonia and influenza	3 days	Call out reason, location, time, age, sex, crude call out number	Respiratory distress, coma, unconsciousness, chest pain, headaches, dizziness, vertigo, fainting, syncope, lying down in public, out-of-hospital cardiac arrest	No, essentially the same dataset as Lin 2021
Wang 2021^*^	Shenzhen, China	All ages (0–17, 18–44, 45–59, ≥ 60)	Time-series 2015–2016	Mean temperature	Seasonality, long-term trend, day of the week, public holidays, relative humidity	7 days	Age, sex, initial diagnosis, crude call out number	All causes and cause-specific	Yes
Williams 2011^†*^	Adelaide, Australia	All ages	Time-series 1993–2009	Minimum and maximum temperatures	Seasonality, long-term trend, day of the week	-	Crude call out number only	All cause	No, used local heatwave definition
Williams 2012^*^	Adelaide, Australia	All ages (≥ 65)	Time-series 1993–2009	Maximum temperature	Within-season variation, long-term trend, day of the week, PM_10_	-	Call out reason, crude call out number	All causes	No, temperature indicator used
Williams 2018^†*^	South Australia, Australia	All ages	Time-series 2000–2015	EHF	Seasonality, long-term trend, day of the week	-	Crude call out number only	All causes	Yes
Williams 2020^*^	Boston, USA	All ages	Time-series 2010–2014	Maximum temperature	Within-season variation, long-term trend, day of the week, O_3_, PM_2.5_	-	Crude call out number only	All causes	No, used local extreme heat event definition
Wu 2021^*^	Shenzhen, China	All ages (0–14, 15–64, ≥ 65)	Time-series 2010–2017	Mean temperature	Seasonality, long-term trend, day of the week, public holidays, relative humidity	21 days	Location, age, sex, symptoms, primary and secondary diagnosis crude call out number	All natural causes	No, no information on the specific values of the 90^th^ and 99^th^ percentiles
Xu 2018^†^	Brisbane, Australia	All ages	Time-series 2008–2015	Multiple temperature indicators	Seasonality, long-term trend, day of the week, PM_10_, NO_2_	7 days	Crude call out number only	All causes	Yes
Yang 2016^*^	Guangzhou, China	All ages	Time-series 2008–2012	Mean temperature	Seasonality, day of the week, public holidays, relative humidity	7 days	Crude call out number only	Renal colic	No, renal colic
Zhan 2018^*^	Shenzhen, China	All ages (0–14, 15–34, 35–64, ≥ 65)	Time-series 2010–2016	Mean temperature	Seasonality, day of the week, public holidays, relative humidity, sunshine duration	28 days	Age, sex, crude call out number, crude call out number	All causes	Yes
Zottarelli 2021^*^	San Antonio, USA	All ages	Case-crossover 2018	Heat index	-	-	Location, crude call out number	All causes	No, temperature indicator used

^†^Studies on the association between heatwave and ambulance service uses

^*^Studies that used temperature data from only one weather monitoring station for each study site

### Meta-analysis

Ten studies were eligible for meta-analysis (Table 3), with nine being of moderate quality. The only high-quality study was included in the meta-analysis on the association between heat and risk of ambulance dispatches for all causes (Sangkharat et al. 2020). For each 5 °C increase in mean temperature, the risk of ambulance dispatches for all causes and for cardiovascular diseases increased by 7% (95% confidence interval (CI): 5%, 10%) and 2% (95% CI: 1%, 3%), respectively (Fig. 2). The pooled statistics suggested that the risk of ambulance dispatches for respiratory diseases did not increase when the mean temperature increased.

**Fig. 4:**
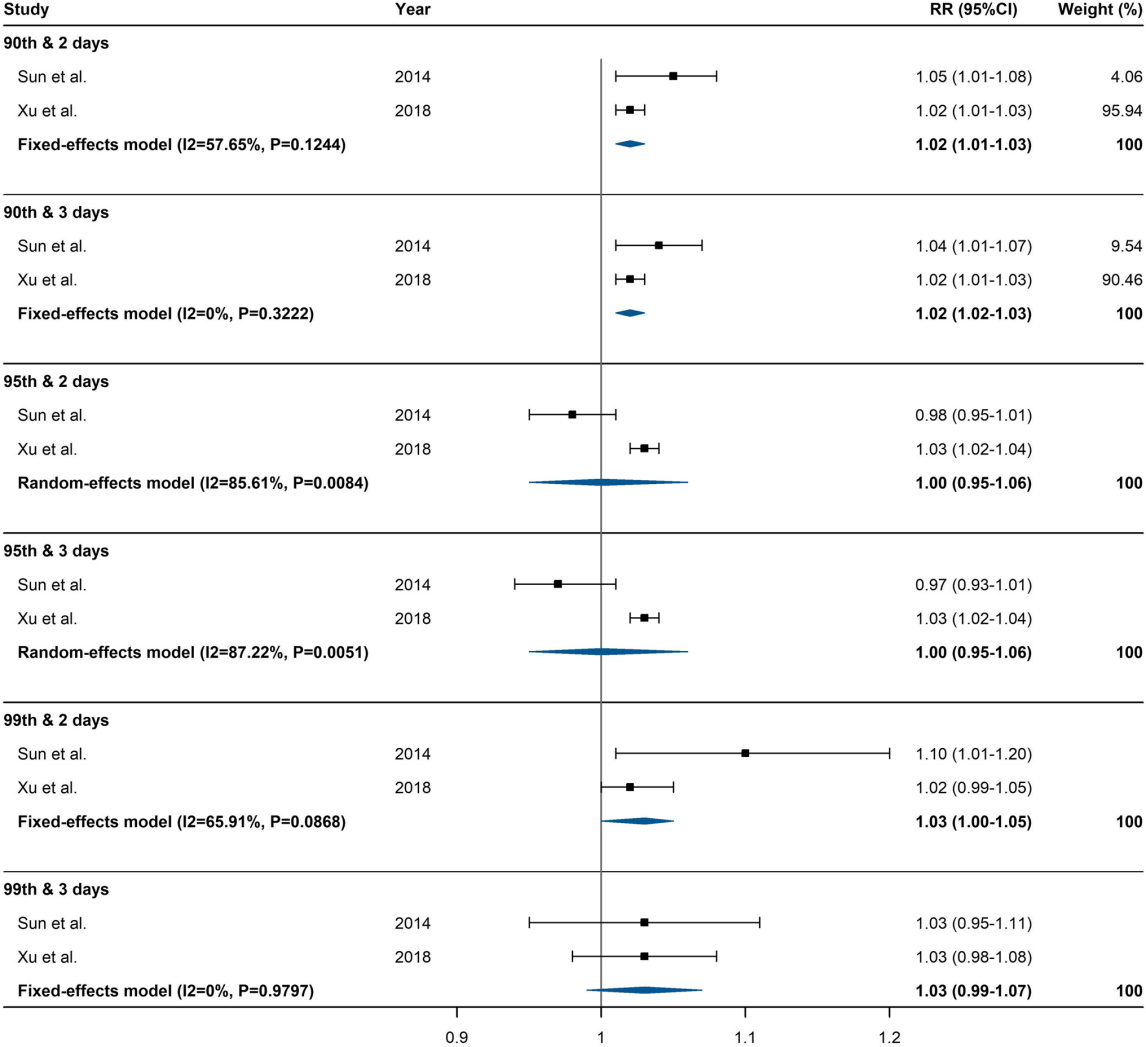
Forest plot for the association between heatwaves (defined by threshold and intensity indicators) and risk of ambulance dispatches

For heatwaves defined by EHF, low-intensity, severe, and extreme heatwaves were associated with 6% (95% CI: 4%, 7%), 7% (95% CI: 5%, 9%), and 18% (95% CI: 12%, 23%) increases in the risk of ambulance dispatches, respectively (Fig. 3). For heatwaves defined by the incorporation of intensity and duration indicators, relatively mild (90th percentile) and very intense (99th percentile) heatwaves were associated with 2% and 3% increases in the risk of ambulance dispatches, respectively (Fig. 4).

### Potential sources of bias in the included studies

We observed two potential sources of bias in the included studies: (1) bias in temperature exposure measurement; and (2) bias in ascertainment of ambulance dispatch causes.

A wide range of temperature indicators have been used in the included studies, including mean temperature, maximum temperature, minimum temperature, apparent temperature, humidex, and heat index. Mean temperature was the most commonly used temperature indicator (*n* = 28). Although spatially refined gridded data on temperature is increasingly accessible, 29 of the 48 studies only used temperature data collected from a single weather monitoring station for each study site (Table 3).

Half of the 48 studies included all-cause ambulance dispatches. A limited number of studies reported specific heat-related events (e.g. heatstroke, dehydration, heat exhaustion) (*n* = 6), cardiovascular (*n* = 17), and/or respiratory (*n* = 11) diseases. However, six studies on cause-specific ambulance dispatches did not publish or published less explicit information on how the ambulance dispatch causes were ascertained (Cui et al. 2020; Lin et al. 2021; Turner et al. 2012, 2013; Wang et al. 2021; Wang et al. 2020).

## Discussion

Studies included in this review suggested a higher risk of ambulance dispatches associated with heat or heatwaves, although 44 of the 48 included studies were of moderate quality. The pooled statistics showed that each 5 °C increase in mean temperature was associated with 7% and 2% increases in the risks of ambulance dispatches for all causes and cardiovascular diseases, respectively. There appeared to be a dose-response relationship between EHF-defined heatwave intensity and the risk of ambulance dispatches.

Exposure to heat can trigger acute life-threatening cardiovascular diseases, such as acute myocardial infarction (Chen et al. 2019) and stroke (Bai et al. 2018). Heat exposure can also trigger acute kidney injury (Borg et al. 2017). The risk of ambulance dispatches increases when the risk of these acute diseases elevates during hot days at a population level. The dose-response relationship between EHF-defined heatwave intensity and the risk of ambulance dispatches, which we observed in the present review, echoed the findings in a recent Australian study which observed a dose-response relationship between heatwave intensity and mortality risk (Xu et al. 2023).

Although we purposely included studies using the two most ideal study designs in quantifying the association between short-term heat exposure and risk of health outcomes (i.e. time-series (Bhaskaran et al. 2013) and case-crossover (Carracedo-Martínez et al. 2010)), the quality of the included studies could have been better if two aspects of the methodology were improved: temperature exposure measurement and ascertainment of ambulance dispatch causes.

### Temperature indicator and temperature exposure measurement

One of the main goals of conducting heat and health research is to facilitate health elements to be incorporated into the development and optimisation of heat adaptation strategies. Different temperature indicators have been used in different countries’ heat early warning systems (HEWS) (sometimes called heat-health action systems). For instance, Australia’s jurisdictional HEWS mainly use the excess heat factor to define heatwaves and trigger heat responses (e.g. the Queensland Heatwave Management Sub-plan (Queensland Health 2019)), and China’s national heatwave alert system uses maximum temperature as the temperature indicator for heatwave definition (China Meteorological Administration 2007). In heat and health research, using the temperature indicator that has been adopted in the national, regional, or local HEWS would facilitate the translation of research outputs into practice. For those countries/regions without HEWS, it is worthwhile conducting research to understand which temperature indicator performs better in predicting heat-related ambulance dispatches (Yu et al. 2011).

Heat exposure could vary within cities (e.g. urban heat island effect), and using temperature data collected from a single monitoring station may underestimate the impact of heat on morbidity if the monitoring station is less representative of heat-vulnerable populations’ exposure (Thomas et al. 2021). With the advent of publicly accessible and well-interpolated temperature data with high spatial resolution (e.g. the 5 km grided temperature data in Australia: https://www.longpaddock.qld.gov.au/silo/, or the worldwide ERA5-Land data at 9 km (Muñoz Sabater 2019) that can be further statistically downscaled to 900 m with KrigR (Byers et al. 2022)), it is possible to use these spatially refined gridded temperature data as an alternative to reduce potential measurement bias. However, we acknowledge that if the spatially refined temperature data was poorly interpolated, it could also cause bias. In the case where publicly accessible and well-interpolated temperature data with high spatial resolution is not available, satellite remote sensing temperature data could also be an option to reduce measurement bias (Xu et al. 2014) (e.g. satellite remote sensing data provided by the US National Aeronautics and Space Administration (NASA): https://ladsweb.modaps.eosdis.nasa.gov/). Because population density may vary across urban and rural areas within cities, Weinberger et al. have used population-weighted temperature derived from spatially refined gridded temperature data (Weinberger et al. 2019), attempting to reduce the temperature measurement bias. In the available studies comparing the health impacts of heat exposure using temperature data collected from limited number of monitoring stations and using spatially refined temperature data, because the models were fitted to the same health outcome and were non-nested, they were unable to quantify the magnitude of the temperature measurement bias. Simulation studies, such as what Wei et al. did in air pollution epidemiology (Wei et al. 2022), will help characterise the magnitude of temperature measurement bias.

### Causes of ambulance dispatches

A main barrier to understanding the potential causal relationship between exposure to heat or heatwaves and the risk of ambulance dispatches is the lack of cause-specific ambulance dispatch data. Some studies included in the present review reported cause-specific ambulance dispatch data. For instance, Campbell et al. used data on ambulance dispatches for cardiovascular, respiratory, renal, diabetic, psychological, direct heat-related, and other heat-related conditions in Tasmania, Australia (Campbell 2021), and they clarified that the records on causes were completed by attending paramedics at the time of, or right after the incident requiring ambulance dispatches.

Unless the patients who used ambulance service have later been hospitalised and gone through diagnostic examinations, it may be hard to accurately ascertain the true causes of ambulance dispatches (particularly in elderly patients who often have multimorbidity (Dobson et al. 2020)). In the present review, the pooled statistics did not suggest an association between heat and the risk of ambulance dispatches for respiratory diseases, but we could not rule out the possibility that respiratory diseases might not have been properly ascertained in the included studies. Data linkage could be an alternative to solve this conundrum. For instance, the information on ICD-codes (international classification of diseases) is generally available in emergency department visit or hospitalisation data. Therefore, linking patients’ ambulance dispatch data with their emergency department visit or hospitalisation data could allow researchers to obtain more accurate information on the causes of ambulance dispatches (Vallmuur et al. 2023).

## Conclusion

The available epidemiological evidence suggests that each 5 °C increase in mean temperature is associated with 7% and 2% increases in the risks of all-cause and cardiovascular ambulance dispatches, respectively. There is a dose-response relationship between EHF-defined heatwave intensity and the risk of all-cause ambulance dispatches. Future studies using well-interpolated or satellite-derived data on spatially refined gridded temperature and linking ambulance data with hospital data may be useful in reducing biases in exposure measurement and health outcome classification.

## Supplementary information

ESM 1 (DOCX 28 kb)
