# Supplementary material for: Heat, heatwaves, and ambulance service use: a systematic review and meta-analysis of epidemiological evidence
Source: Int J Biometeorol. 2023 Jul 27;67(10):1523–42. doi: 10.1007/s00484-023-02525-0 (PMC10457246; doi:10.1007/s00484-023-02525-0)
Supplement: Supplementary file 1 — (DOCX 28 kb) [file 484_2023_2525_MOESM1_ESM.docx]

**Supplementary Materials**

**Heat, heatwaves, and ambulance service use: A systematic review and meta-analysis of epidemiological evidence**

**Supplementary 1. Search strategy (between categories was searched with AND)**

| Category 1: Heat term (searched with OR) | Category 2: Ambulance term (searched with OR) | Category 3: Health term (searched with OR) | Category 4: Searched with NOT |
| --- | --- | --- | --- |
| Hot | Ambulance | Health | COVID-19 |
| Heat | Emergency service | Morbidity | Vaccination |
| Heatwave | Emergency medical technician | Mortality | Influenza |
| Heat wave | Paramedic | Comorbidity | Pre-cooling |
| Hot day | Emergency medical service | Health condition | Pre cooling |
| Hot weather | Ambulance call out | Comorbidities | Meta-analysis |
| Hot temperature | Ambulance call-out | Death | Systematic review |
| Extreme heat | Ambulance dispatch | Illness | Randomized control trial |
| Extreme hot day | Emergency health service | Emergency department visit | Non randomized control trial |
| Extreme temperature | Emergency medical treatment | Hospital admission | Rapid review |
| Extreme hot weather | EMS | Health service utilization | Narrative review |
| Temperature | EMT | Health service | Scoping review |
| Hot temperature |  | Hospitalisation | Storm |
| High temperature |  |  | Coronavirus |
| Season |  |  |  |
| Seasonal |  |  |  |
| Seasonal variation |  |  |  |
| Summer |  |  |  |
| Warm weather |  |  |  |
| Warm day |  |  |  |
| Extreme environment |  |  |  |

**Supplementary 2. Quality assessment tools**

Time-series design quality assessment tool

| Question | Responses |
| --- | --- |
| Selection | |
| Representativeness of the sample | |
|  | Truly representative of the target geographical area (entire area or random sample)*** |
|  | Somewhat representative of the target geographical area (non-random sampling)** |
|  | High likelihood of being unrepresentative of the target geographical area (e.g., convenience sample)* |
|  | No description of the derivation of the sample |
| Exposure | |
| Ascertainment of ambient temperature exposures defined by the authors (e.g., defined by temperature thresholds, or by geographic regions) | |
|  | Secure instrumental record (e.g., thermometers in weather stations, satellite data, established heatwave/cold bell)*** |
|  | Structured interview** |
|  | Written self-report* |
|  | No description |
| The size of the regional unit in the temperature exposure assignment | |
|  | Smaller than (not including) city in the U.S., county in China, or the equivalent in other countries; smaller than 15km (not including) in grid side length regarding grid temperature exposures; or smaller than (not including) 8.5km regarding distance from temperature monitors*** |
|  | Equal to city in the U.S., county in China, or the equivalent in other countries; ranging from 15km (including) to 35km (including) in grid side length regarding grid temperature exposures; or ranging from 8.5km (including) to 20km (including) regarding distance from temperature monitors** |
|  | Larger than (not including) city in the U.S., county in China, or the equivalent in other countries; larger than (not including) 35km in grid side length regarding grid temperature exposures; or larger than (not including) 20km regarding distance from temperature monitors* |
|  | No description or the regional unit is unknown |
| Comparability | |
| The study controls for long-term trends, seasonality, and day of week (when the outcome is daily). | |
|  | Yes* |
|  | No |
| The study controls for at least one other important potential confounder (air pollutants). | |
|  | Yes* |
|  | No |
| Outcome | |
| Assessment/specificity of outcome | |
|  | Ambulance data limited to heat related codes only AND accidental causes removed*** |
|  | Ambulance data limited to heat related codes only OR accidental causes removed** |
|  | All causes followed, or specific causes (e.g. CVD, stroke)* |
|  | No description of specificity |
| Quality assessment | |
| Overall score (out of 14 stars) | |
|  | Low 0-6 stars (0-49%) |
|  | Moderate 7-11 stars (50-84%) |
|  | High 12-14 stars (85-100%) |
| Comments: |  |

Case-crossover design quality assessment tool

| Question | Responses |
| --- | --- |
| Selection | |
| 1. Is the case definition adequate? | |
|  | Yes, with independent validation (e.g., >1 person/record/time/process to extract information, or reference to primary record sources such as insurance records, medical/hospital records, or death certificates)** |
|  | Yes, e.g., record linkage or based on self-reports, with no reference to primary record* |
|  | No description |
| 2. Representativeness of the cases | |
|  | Consecutive or obviously representative series of cases (e.g., all eligible cases with outcome of interest over a defined period of time, all cases in a defined catchment area, all cases in a defined hospital or clinic, or an appropriate sample of those cases [e.g., random sample]) * |
|  | Potential for selection biases (i.e., does not satisfy requirement in part (a) or not stated) |
| 3. Selection of controls | |
|  | Community controls (e.g., same source population as cases and would be cases if had outcome; representative of the source population [e.g., a random sample of the source population])** |
|  | Hospital controls (or other controls that are not representative of the source population)* |
|  | No description |
| 4. Definition of controls | |
|  | No history of disease outcome (if cases have new [not necessarily first] occurrence of outcome, then controls with previous occurrences of outcome of interest should not be excluded)* |
|  | No mention of history of disease outcome |
| Comparability | |
| 5. The study controls for at least one other important potential confounder (air pollutants). | |
|  | Yes* |
|  | No |
| Exposure | |
| 6. Ascertainment of ambient temperature exposures defined by the authors (e.g., defined by temperature thresholds, or by geographic regions) | |
|  | Secure instrumental record (e.g., thermometers in weather stations, satellite data, established heat waves/cold spells)**** |
|  | Structured interview where blind to case/control status*** |
|  | Interview where not blind to case control status** |
|  | Written self-report* |
|  | No description |
| 7. Same method of ascertainment of ambient temperature exposures for cases and controls. | |
|  | Yes* |
|  | No |
| 8. The size of the regional unit in the temperature exposure assignment. | |
|  | Smaller than (not including) city in the U.S., county in China, or the equivalent in other countries; smaller than 15km (not including) in grid side length regarding grid temperature exposures; or smaller than (not including) 8.5km regarding distance from temperature monitors*** |
|  | Equal to city in the U.S., county in China, or the equivalent in other countries; ranging from 15km (including) to 35km (including) in grid side length regarding grid temperature exposures; or ranging from 8.5km (including) to 20km (including) regarding distance from temperature monitors** |
|  | Larger than (not including) city in the U.S., county in China, or the equivalent in other countries; larger than (not including) 35km in grid side length regarding grid temperature exposures; or larger than (not including) 20km regarding distance from temperature monitors* |
|  | No description or the regional unit is unknown |
| Quality Assessment | |
| Overall score (out of 15 stars) | |
|  | Low 0-7 (0-49%) |
|  | Moderate 8-12 (50-84%) |
|  | High 13-15 (85-100%) |
| Comments: |  |

**Supplementary 3. Definitions of the temperature indicators used in the included studies**

| **Temperature indicator** | **Temperature indicator definition** |
| --- | --- |
| Air temperature | A measure of how hot or cold the air is. When people use maximum temperature, mean temperature, or minimum temperature, it normally refers to air temperature (unless otherwise stated). |
| Apparent temperature | The temperature perceived by humans, caused by a combined effects of air temperature, relative humidity and wind speed. |
| Compound heat | A hot day followed by a hot night. For instance, in the study of He et al. (DOI: 10.1016/j.accre.2021.09.001) which was included in the present review paper, compound heat was defined as a day that daily maximum and minimum temperatures are higher than the 90th percentile for the temperature ranges |
| Excess heat factor | Excess heat factor (EHF) is an index based on a three-day-averaged daily mean temperature, and it is mainly used to capture heatwave intensity. The EHF was first introduced by John Nairn and Robert Fawcett (DOI: 10.3390/ijerph120100227), and has been used in multiple jurisdictional heat early warning systems in Australia |
| Heat Index | Heat index is similar to apparent temperature as it refers to the temperature perceived by humans. However, it is a temperature indicator which combines air temperature and relative humidity. Heat index is primarily used in the USA. |
| Humidex | Humidex is a temperature indicator which also refers to the temperature perceived by humans. It combines air temperature and dew point temperature (DPT, a humidity indicator), and it is primarily used in Canada. |
